# Mode I Stress Intensity Factor Solutions for Cracks Emanating from a Semi-Ellipsoidal Pit

**DOI:** 10.3390/ma17194777

**Published:** 2024-09-28

**Authors:** Hasan Saeed, Robin Vancoillie, Farid Mehri Sofiani, Wim De Waele

**Affiliations:** Soete Laboratory, Department of Electromechanical, Systems and Metal Engineering (EMSME), Faculty of Engineering and Architecture, Ghent University, Technologiepark 46, 9052 Zwijnaarde, Belgium; hasan.saeed@ugent.be (H.S.); vancoillierobin@gmail.com (R.V.); farid.mehri.sofiani@gmail.com (F.M.S.)

**Keywords:** stress intensity factor, semi-ellipsoidal pit, finite element analysis, displacement extrapolation method, machine learning

## Abstract

In linear elastic fracture mechanics, the stress intensity factor describes the magnitude of the stress singularity near a crack tip caused by remote stress and is related to the rate of fatigue crack growth. The literature lacks SIF solutions for cracks emanating from a three-dimensional semi-ellipsoidal pit. This study undertakes a comprehensive parametric investigation of the Mode I stress intensity factor ${(K}_{I})$ concerning cracks originating from a semi-ellipsoidal pit in a plate. This work utilizes finite element analysis, controlled by Python scripts, to conduct an extensive study on the effect of various pit dimensions and crack lengths on $K_{I}$. Two cracks in the shape of a circular arc are introduced at the pit mouth perpendicular to the loading direction. The $K_{I}$ values are calculated using the displacement extrapolation method. The effect of normalized geometric parameters pit-depth-to-pit-width (a/2c), pit-depth-to-plate-thickness (a/t), and crack-radius-to-pit-depth (R/a) are investigated. The crack-radius-to-pit-depth (R/a) is found to be the dominating parameter based on correlation analysis. The data obtained from 216 FEA simulations are incorporated into a predictive model using a k-dimensional (k-d) tree and k-Nearest Neighbour (k-NN) algorithm.

## 1. Introduction

Fatigue failures in steel structures and components, where pit formation precedes crack development, remain a significant challenge [1,2,3] in industries such as offshore renewable energy, aerospace, and oil and gas. Surface pits induce stress concentration, potentially serving as starting points for fatigue cracks under dynamic loads, potentially reducing the lifespan of components compared to those without pits. The role of pits as crack initiation spots is linked to the concept of a stress concentration factor (SCF), which quantifies the amplified stress at geometrical discontinuities under specific loading conditions. This phenomenon has encouraged extensive research into SCFs associated with pits [4,5,6,7,8,9,10,11]. However, once a crack is initiated, the concept of SCF is no longer valid. Then, a stress intensity factor (SIF) solution is needed to quantify the stresses at the crack tip and to evaluate the fatigue crack growth and potential unstable failure. Analytical solutions to calculate the SIF for a crack emanating from a pit are currently not available in the literature.

Several studies [12,13,14,15] employed a conservative approach in estimating the crack growth of a pitted specimen. They assume that at the point of transition from a pit to a crack, the pit can be treated as equivalent to a crack. Replacing the volumetric pit with a two-dimensional semi-elliptical crack will clearly lead to conservative SIF values.

**Figure 1:**
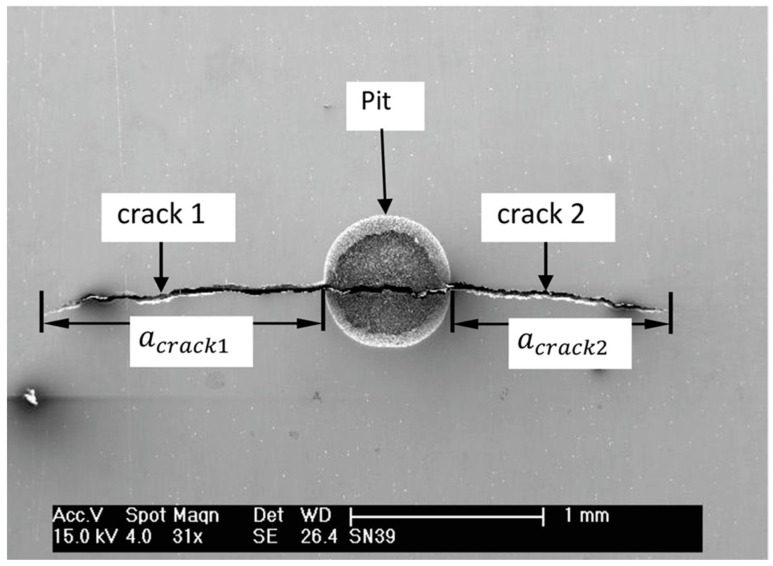
SEM image of propagating fatigue crack that initiated from the pit mouth (reproduced with permission from [16]).

Several experimental studies indicate that fatigue cracks typically originate at the pit mouth [16,17,18]. A study involving API 5L X65 steel, where specimens were pre-pitted electrochemically and subjected to fatigue loading, identified instances of cracks being initiated from the mouth of the pits [16]. This location was also confirmed using SEM analysis in (see Figure 1) a study involving artificially generated corrosion pits on a 12% Cr martensitic steel steam turbine blade subjected to fatigue load [17]. In a study employing computed tomography for 3D imaging of pits and cracks in 3NiCrMoV disc steel, it was observed that in the majority of the specimens tested, the crack initiation was closer to the pit mouth than to the pit bottom [18]. In contrast, in a study on stress corrosion cracking (SCC), ‘finger-like crack’ sites were observed, using optical microscopy, at the bottom of the pit [19].

In an effort to reduce the conservatism of earlier studies, Rokhlin et al. [20] applied the SIF solution for a corner crack at the edge of a through-thickness hole in a plate [21] during the initial phase of crack growth, before transitioning to the semi-elliptical crack model. This approach, referred to as the ‘two-step crack growth model’, was further used by Schönbauer et al. [17] and Fatoba et al. [16] in the estimation of the fatigue crack growth rate (FCGR) for cracks initiating from the mouth of the pit.

To understand the effects of the three-dimensional geometry of pits on crack growth, Turnbull et al. [22] and Wang et al. [23] used a 3D finite element model (FEM) to calculate the stress intensity factor for a crack developing from a pit in a cylindrical component and cast iron pipes. In his study, the contour integral method is used to develop the $K_{I}$ solution for a crack initiating from the pit mouth. It is the only study (at the moment of writing) in which $K_{I}$ values were calculated from an FEM simulation for a 3D pit with cracks that emanated from the mouth of the pit in a limited configuration. In another study, Zhang et al. [24] investigated the stress intensity factor of a crack present at the bottom of the pit in a plate, in which he also used a contour integral analysis for the investigation of an SIF.

In computational LEFM, commonly used methods for calculating SIF values include the displacement extrapolation method [25,26] and the J-integral method [23,27]. These methods relate an SIF to physical properties like displacement, stress, or energy around the crack tip, ascertained through finite element analysis. Studies based on energy principles show higher accuracy compared to techniques that extrapolate stress or displacement fields near the crack tip [28,29,30]. Some studies based on displacement extrapolation show that a precise numerical model of the area surrounding the crack tip is necessary for the accurate determination of an SIF [26,31]. Qian et al. [29] stated that 3D models yield more precise outcomes compared to 2D models. The choice of element type has also been investigated for its impact on accuracy. According to Han et al. [30], employing quadratic elements enhances the precision of SIF calculations over linear elements. Similarly, investigations into the efficacy of standard linear and quadratic element types, versus specialized singular elements, like quadrilateral and triangular quarter-point elements at the crack tip, revealed that singular elements result in a higher accuracy for both displacement extrapolation [31,32,33] and J-integral methods [30,33]. Both the J-integral method and the displacement extrapolation method for SIF calculation have their pros and cons. A comparison between these two methods is listed in Table 1.

The displacement extrapolation method is applied in this study for its ease in programming and integration into a parametric FEA framework. In this study, a 3D FEM is developed for a pit with cracks emanating from the mouth of the pit. A parametric investigation is performed for the impact of various pit configurations and crack lengths on the $K_{I}$ values at the crack tips. The analyses reported in the literature are restricted to a limited number of specific configurations and do not provide a generic solution for a crack emanating from the mouth of a pit in a plate. One study [22] focused on a crack that emanated from the pit mouth in a cylindrical component. Another study [24] focused on a crack at the bottom of a pit in a plate. Our research addresses this gap by systematically investigating the effects of pit depth-to-width ratios (a/2c), pit depth-to-plate thickness ratios (a/t), and crack radius-to-pit depth (R/a) configurations on the stress intensity factor for a crack emanating from the mouth of a pit in plate component.

This study employed a full factorial design of experiments (DOE) approach to investigate the influence of geometric parameters and their interactions on the stress intensity factor $K_{I}$ in numerical simulations. All parameters were analyzed at six levels to effectively determine their impacts on the $K_{I}$. The use of dimensionless geometric parameters (pit depth-to-pit width a/2c, pit depth-to-plate thickness a/t and crack radius-to-pit depth R/a) and shape factor reflects the underlying principles of dimensional analysis and similarity, as these ratios allow us to model the stress intensity that can be scaled across different pit and crack sizes.

Polynomial regression has been commonly used to model the relationship between geometric parameters and stress intensity factors, allowing the capture of non-linear behaviours. However, it has limitations such as underfitting, where a low-degree polynomial misses important patterns, and overfitting, where a high-degree polynomial captures noise instead of the true trend, leading to poor generalization. To overcome these issues, recent studies have shifted towards machine learning algorithms. In this study, the obtained results are post-processed using a K-dimensional tree to allow calculating $K_{I}$ solutions for a range of pit configurations and crack lengths. The $K_{I}$ solutions presented in this paper are applicable exclusively under conditions where LEFM concepts are valid.

This paper is structured as follows. The next section describes the development of the 3D FEM, and its integration with Python for generating and post-processing a large number of FEMs. The following section explains the methodology for calculating $K_{I}$ values using the displacement extrapolation method. The subsequent section presents the results of this parametric study, illustrating the influence of geometric parameters on $K_{I}$ values. Finally, the dataset is used to develop a surrogate model for $K_{I}$ values using a K-dimensional tree and a user-friendly GUI.

## 2. Finite Element Model

**Figure 2:**
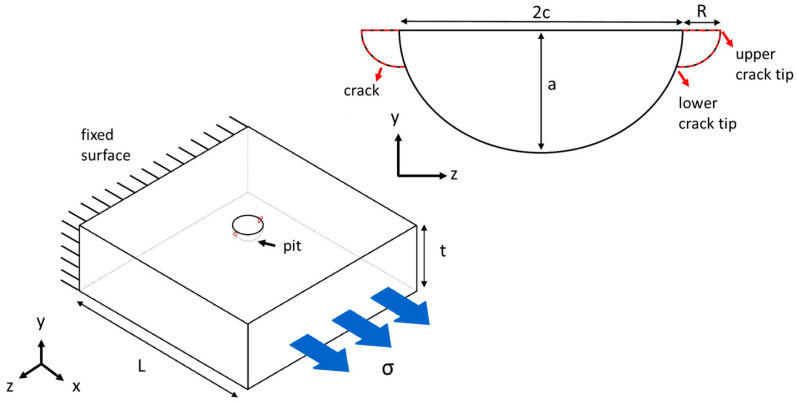
Schematic representation of the geometrical model of a pitted plate with emanating cracks for finite element analysis.

The schematic in Figure 2 depicts the 3D geometrical model and its dimensions. This study focuses on pits with a circular surface profile and a semi-ellipsoidal cross section. The diameter of the pit mouth is represented by 2c. The ratio of crack depth to plate thickness a/t is assumed to be smaller than 0.1, to realistically simulate pits in thick-walled structures. The ratio of the length of the plate to the pit width, L/2c, is higher than 15, to avoid boundary effects in all models [34]. A uniaxial tensile stress (σ) is applied remotely at one side of the plate and parallel to the plate length, while the other side is assigned a fixed boundary condition.

The cracks considered in this study are quarter circular with a radius of r, emanating from the pit mouth and perpendicular to the direction of the applied stress. A parametric study is performed for six values of a/2c, six values of R/a, and six values of a/t, (see Table 2), yielding a total number of 216 models. The parameter a/2c, ranging from 0.1 to 2.0, represents pits that range from shallow and wide to deep and narrow. The ratio R/a, spanning from 0.01 to 0.5, describes crack sizes from very small compared to the pit size to half the pit depth. The a/t ratio, also ranging from 0.01 to 0.5, reflects pit sizes corresponding to periods of early growth up to longer term evolutions. Six values for each parameter are selected to create a dataset suitable for detailed statistical analysis, allowing for the identification of trends within these ranges. The displacement extrapolation method is employed for the calculation of $K_{I}$, as will be discussed in Section 3.

**Figure 3:**
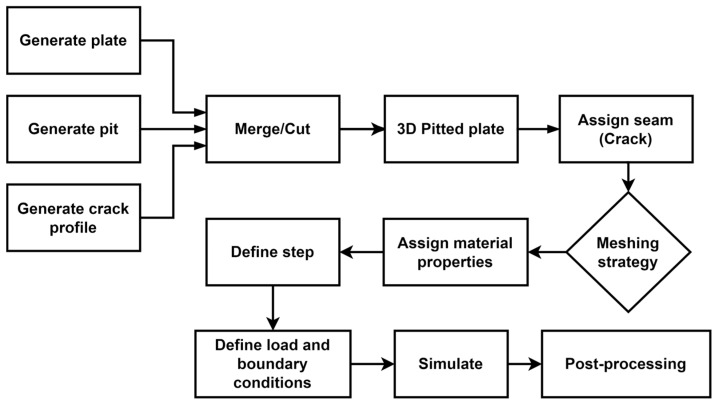
Flowchart of the script used to automate the finite element modelling and analysis.

Python scripts are developed and integrated with finite element software ABAQUS^®^ CAE 2023 to automate the pre- and post-processing steps. The script is architected to create a parametrized model of a semi-ellipsoidal pit in a plate subjected to tensile load. Figure 3 provides a flowchart of the script.

**Figure 4:**
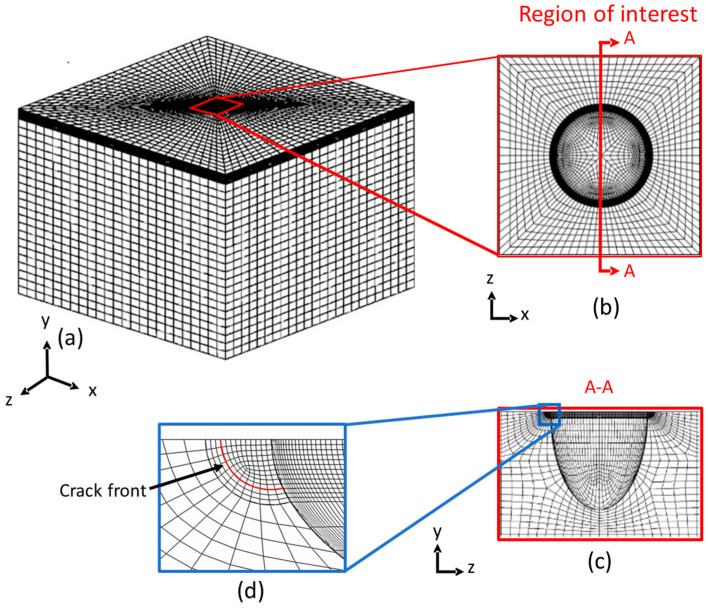
Representation of a hexahedral FE mesh; (**a**) prismatic view of the plate with pit, (**b**) a close-up view of the pit, (**c**) a cross-sectional view of the pit with a crack and (**d**) a close-up view of the crack front at the pit mouth.

To develop a parametrized geometrical model, three different objects are merged to create a 3D pitted plate. The semi-ellipsoidal pit is cut from the plate, after which the cracks’ edges are introduced at the mouth of the pit. A seam (overlapping duplicate nodes) is assigned to the cracks’ edges to define infinitely sharp cracks. The model edges are partitioned and seeded to obtain a qualitative mesh. Two mesh refinement strategies with quadratic elements (a combination of C3D20 hexahedral and C3D10 tetrahedral) are used. The C3D20 element is a 20-node hexahedral element, shaped like a brick, and is preferred for its higher accuracy in modelling straight-edged geometries. However, it is more computationally demanding due to its increased number of nodes. On the other hand, the C3D10 element is a 10-node tetrahedral element, well suited for simulating complex 3D geometries and capable of handling large deformations. Some of the studied pit configurations can be not meshed using hexahedral elements due to their complex geometry and are meshed using tetrahedral elements. The combination of both strategies allows modelling a wide spectrum of pit configurations. A transition from the fine mesh at the region of interest to a coarser mesh is designed, to reduce the computational time. Figure 4 shows a meshed finite element model, emphasizing the areas in proximity to the cracks and around the pit. The modulus of elasticity and Poisson’s ratio are taken as E  = 207 GPa and ν = 0.3, representing low-carbon steel.

## 3. Displacement Extrapolation Method

**Figure 5:**
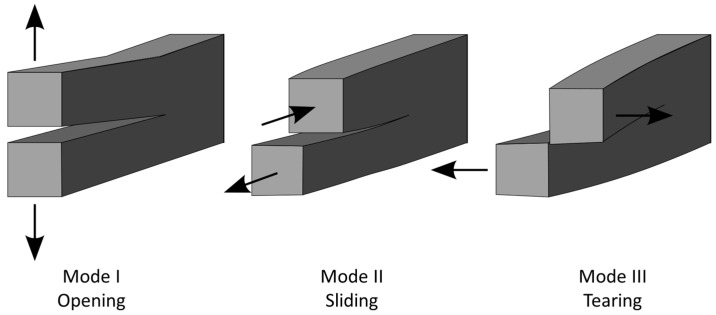
Three primary modes of crack propagation encountered in fracture mechanics (adapted from [35]).

In this study, we exclusively focus on Mode I stress intensity factor ($K_{I}$) calculations. Mode I, also known as the opening mode, is the most critical and common mode of fracture in materials under tensile stress. As shown in Figure 5, focusing on Mode I allows for a more straightforward analysis using FEA, as it involves simpler boundary conditions and stress states compared to mixed-mode or Mode II (sliding mode) and Mode III (tearing mode) fractures.

The displacement extrapolation method is implemented to derive the $K_{I}$ values. This method uses nodal displacement data near the crack tip to estimate the $K_{I}$ value, bypassing the need for direct analytical solutions of stress and displacement fields around singularities. The essential prerequisite of the method is that the finite element analysis provides accurate displacement results in the neighbourhood of the crack tip [25]. These displacements are typically measured in a polar coordinate system, i.e., radial displacement $(u_{r}$) and angular displacement ${(u}_{\theta })$. When the radial coordinate (r) equals the crack radius (R) and the angular coordinate θ equals π, the displacements are evaluated along the crack edge. Consequently, $u_{r}$ becomes redundant, and only $u_{\theta }$ perpendicular to the radial direction is required. The equation obtained from the derivation presented in [36] for plane stress conditions is given in Equation (1). This plane stress assumption is justified as the upper and lower crack tips are situated at a free surface.

(1) $$K_{I}=\frac{E}{4}\sqrt{\frac{2\pi }{r}}u_{\theta }$$

**Figure 6:**
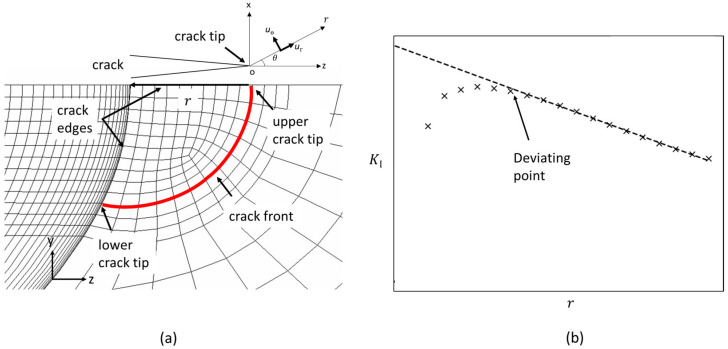
(**a**) Crack tip mesh with illustration of the reference frame in the node for which the SIF is calculated, (**b**) Illustration of the displacement extrapolation method to determine the SIF value at the crack tip.

In this analysis, the $u_{\theta }$ and $K_{I}$ value are calculated at nodes along the crack edges. The algorithm discards the results for nodal points close to the crack tip and identifies the linear region in the dataset by analyzing the slopes between successive data points. Starting from the point that is most remote from the crack tip, it iteratively calculates the slope between each pair of adjacent points and checks for consistency. Consistent slopes signify the linear portion, while significant deviations indicate the transition away from linearity. Once the linear portion is identified, linear regression is performed on this subset to determine the intercept as the value of $K_{I}$ at the crack tip (see Figure 6b).

**Figure 7:**
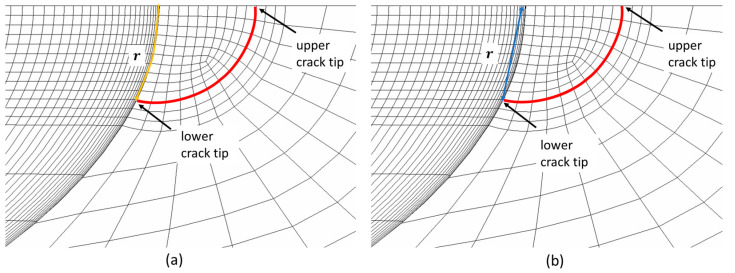
Illustration of the radial path used to calculate $K_{I}$: (**a**) arc length (yellow) and (**b**) projected length (blue) perpendicular to the crack front (red) for the lower crack tip.

In the literature [36], the r value used in Equation (1) to calculate $K_{I}$ is defined as the distance measured perpendicular to the crack front. However, this becomes complex for curved paths like those depicted in Figure 7, where nodes do not lie directly on the intended trajectory. To overcome this challenge, two methods are proposed. The first method substitutes the radial distance with the arc length, as illustrated in Figure 7a. The second method involves projecting the coordinates of each node onto the radial axis, as illustrated in Figure 7b. This second method is preferred because it is aligned with the original definition. It also tends to provide a more conservative $K_{I}$ estimate due to the slightly smaller R values.

A mesh convergence study is performed to determine the optimal element sizes for both hexahedral elements and tetrahedral elements. Alongside the accuracy of results, computational time is considered as an additional criterion to aid in selecting the most appropriate element size. A model with geometric ratios a/2c = 0.66, R/a = 0.65, and a/t = 0.1 is used to perform the mesh convergence study for both hexahedral (C3D20) and tetrahedral (C3D10) elements. The refinement along the quarter circular partitions (i.e., the crack front) is studied, while simultaneously refining the free surface edges (i.e., crack opening edges) to ensure an acceptable aspect ratio of less than 10 [37] for faster convergence of calculations. The simulations are performed on a processor with the following specifications: Intel^®^ Xeon^®^ Gold 6152 CPU, 2.1 GHz, and 256 GB RAM. Furthermore, a maximum of 90% of the preprocessor and analysis memory is used, and 14 CPUs are used for the calculations.

**Figure 8:**
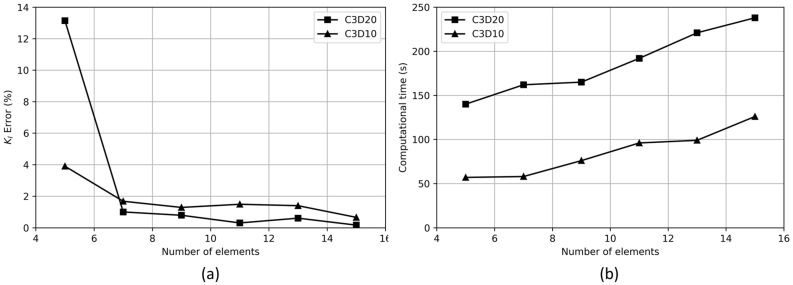
(**a**) $K_{I}$ error (%) and (**b**) computational time, versus number of elements at crack for a pit with a/2c = 0.66, R/a = 0.65, and a/t = 0.1.

Figure 8 illustrates the outcomes of the mesh convergence study for various numbers of elements along the crack opening edges. In this convergence study, the $K_{I}$ error (expressed as a percentage) is calculated by comparing the $K_{I}$ value derived for a certain number of elements to that obtained from a very finely discretized mesh comprising 31 elements along the crack edges, which is considered as the benchmark.

The combination of high accuracy and faster computing time for the tetrahedral element is observed to be optimal for nine elements. The consideration of computational time is crucial, given the need to conduct and post-process 216 simulations within a practical time frame. The $K_{I}$ error for the tetrahedral element is less than 2% for 9 elements, which is considered acceptable. The convergence study also highlights that the 20-node hexahedral element offers greater accuracy compared to the 10-node tetrahedral element, though this increased accuracy comes with much higher computational costs.

In the following section, the displacement extrapolation method is applied to the data derived from a series of FE simulations. It entails analyzing output files to ascertain relative displacements and distances at individual data points, and when utilizing these data, the value of $K_{I}$ is calculated. Subsequently, the $K_{I}$ values determined at these points are extrapolated to estimate the value of $K_{I}$ at both the upper and lower crack tips.

## 4. Numerical Results and Analysis

**Figure 9:**
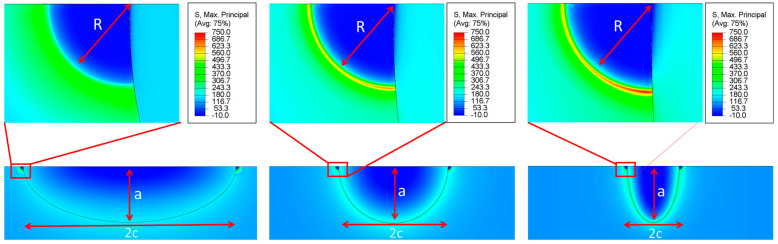
Stress plot of three different pits configurations: a/2c = (0.25, 0.5, 1.0), R = 0.1 mm, a = 1.5 mm at applied stress of 100 MPa.

This section discusses the results of this parametric study of the effects of pit and crack geometrical parameters on the Mode I stress intensity factor ($K_{I})$. The pits investigated in this study are classified on the basis of their geometric ratios. The influence of normalized geometrical parameters on the Mode I Stress intensity factor $K_{I}$ is extensively analyzed for different configurations of semi-ellipsoidal pits with cracks emanating from their mouths (see Table 2). A series of 216 simulations is performed, for which two quarter-circular cracks are modelled at the pit mouth, oriented perpendicular to the direction of the applied uniaxial stress with a value of 100 MPa. Figure 9 illustrates the maximum principal stress distribution around semi-ellipsoidal pits with different a/2c ratios under an applied uniaxial tensile stress of 100 MPa.

These visualizations show how changes in the pit depth-to-width ratio influence stress concentration. In the first configuration, where a/2c= 0.25, the pit is shallow and wide. This results in a lower and more evenly distributed stress concentration around the pit edge. The stress concentration near the crack tip is relatively modest, indicating a less severe stress concentration for shallow pits. The second configuration, with a/2c= 0.5, shows a balanced depth-to-width ratio. The stress concentration increases compared to the shallow pit configuration. The stress levels near the crack tip are higher, signifying an increase in stress intensity as the pit depth becomes more comparable to its width. In the third configuration, where a/2c= 1.0, the pit is deep and narrow. The stress concentration near the lower crack tip is significantly higher, illustrating that deeper pits result in more pronounced stress concentrations, especially near the crack tips. Notably, the lower crack tip exhibits a slightly higher stress intensity compared to the upper crack tip.

**Figure 10:**
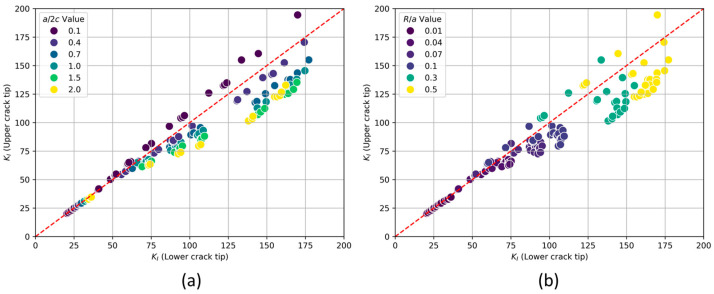
Scatter plot of $K_{I}$ (MPa.√mm) at upper and lower crack tips. Results are organized for (**a**) groups of a/2c values and (**b**) groups of R/a values.

To validate this observation across all configurations, a scatter plot was generated allowing for a comprehensive comparison of the $K_{I}$ values at both the upper and lower crack tips (see Figure 10). The results are shown in groups of constant values of the parameters a/2c and R/a, which help to identify consistent patterns in these results.

Each plot shows the relationship between the $K_{I}$ at the lower and upper crack tips. The diagonal red dashed line represents the equality line where the $K_{I}$ for the lower and upper crack tips would be equal. When a/2c is 0.1 (see Figure 10a), the $K_{I}$ at the upper crack tip tends to be higher than at the lower crack tip. This is indicated by points lying above the red dashed line. As a/2c increases, the points begin to shift below the red dashed line, indicating that the $K_{I}$ at the lower crack tip becomes higher. This shows that increasing the a/2c ratio changes the dominance of stress intensity from the upper to the lower crack tip. Figure 10b presents the relationship between $K_{I}$ at the lower and upper crack tips with varying R/a values. For low R/a values (0.01–0.07), the $K_{I}$ at both crack tips are clustered closer to the red dashed line, suggesting that the $K_{I}$ at both crack tips are nearly equal. There is a slight tendency for some points to lie above the red dashed line, indicating that the $K_{I}$ at the upper crack tip might be marginally higher when a/2c is equal to 0.1. As the R/a value increases, the overall $K_{I}$ values tend to increase. This is evident from the higher concentration of points with larger $K_{I}$ values in the higher R/a ranges.

### 4.1. Evaluation for Upper Crack Tip

**Figure 11:**
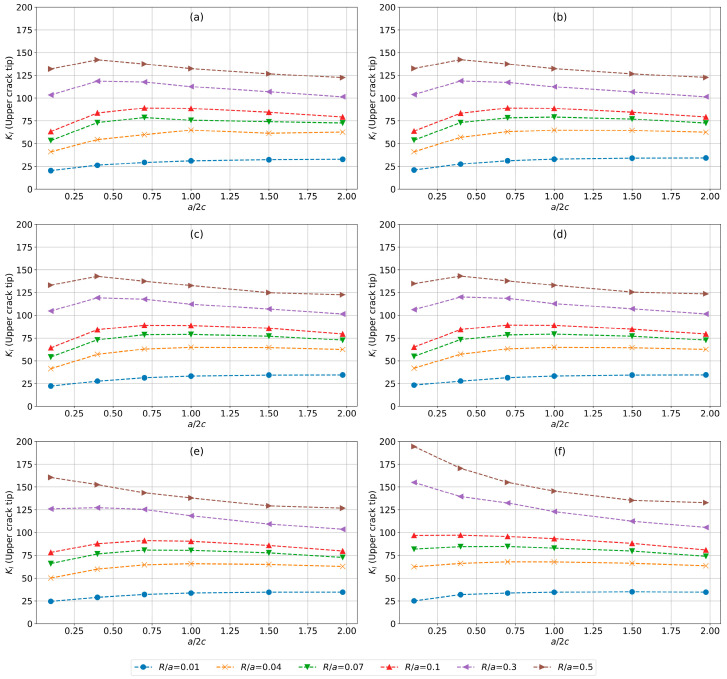
Evolution of $K_{I}$ (MPa.√mm) for the upper crack tip for a/t equal to (**a**) 0.01, (**b**) 0.04, (**c**) 0.07, (**d**) 0.1, (**e**) 0.3, and (**f**) 0.5, and varying a/2c and R/a values.

Each plot of Figure 11 represents a different a/t ratio, increasing from 0.01 in plot Figure 11a to 0.5 in plot Figure 11f. The individual plots explore the effect of the a/2c ratio (with values ranging from 0.1 to 2.0), and varying crack-radius-to-pit-depth ratios R/a (with values ranging from 0.01 to 0.5), to provide a comprehensive understanding of their effect on $K_{I}$.

In the plots for the lower a/t ratios (0.01–0.1) shown in Figure 11a–d, $K_{I}$ values initially increase as a/2c rises from 0.1 to approximately 0.5–1.0, after which they largely stabilize or show a very modest decrease. This increase and plateauing effect are more pronounced for smaller R/a ratios. The curve for R/a= 0.01 stands out, showing a consistently lower magnitude of $K_{I}$ across all values of a/2c. This shows that when the crack radius is very small compared to the pit depth, the stress intensity factor remains lower, regardless of the a/2c ratio. The second group, consisting of R/a values of 0.04, 0.07, and 0.1, displays a more pronounced increase in $K_{I}$ as a/2c rises from 0.1 to about 1.0, after which the values either stabilize or slightly decrease. This indicates a moderate increase in stress intensity with an increasing crack size relative to the pit depth. The third group, which includes R/a values of 0.3 and 0.5, shows a slightly different trend. These curves also increase initially but tend to peak earlier and then decline more noticeably as a/2c increases.

As a/t increases (see Figure 11e,f), the increase in $K_{I}$ becomes more pronounced in terms of magnitude. For smaller R/a, a similar trend is seen across all the a/t ratios. For higher R/a ratios (0.3–0.5), a higher magnitude of $K_{I}$ is notable followed by a decline as the a/2c ratio increases, further reducing peak intensity.

### 4.2. Evaluation for Lower Crack Tip

**Figure 12:**
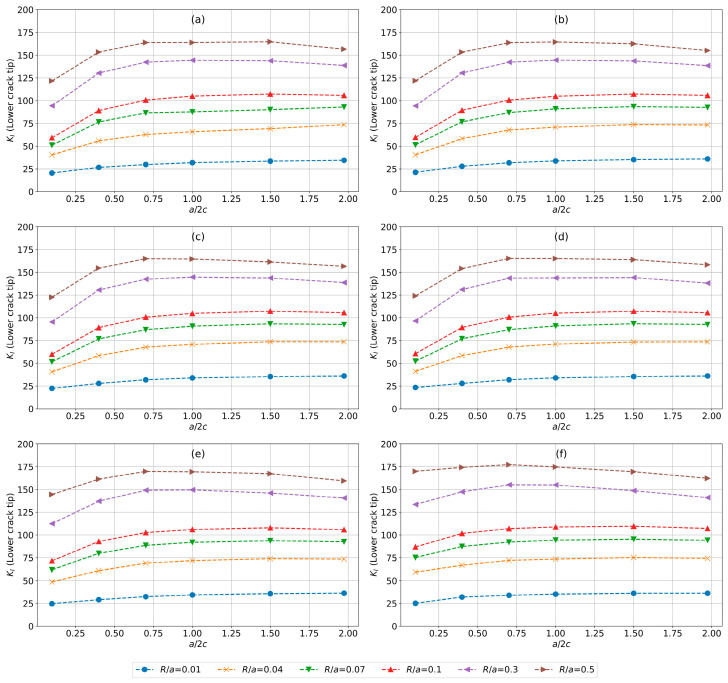
Evolution of $K_{I}$ (MPa. √mm) for the lower crack tip for a/t equal to (**a**) 0.01, (**b**) 0.04, (**c**) 0.07, (**d**) 0.1, (**e**) 0.3, and (**f**) 0.5, and varying a/2c and R/a values.

Similar to the upper crack tip, the sequence of plots from Figure 12a–f shows the influence of varying a/2c, R/a, and a/t ratios on $K_{I}$ at the lower crack tip.

The trends in lower-bound $K_{I}$ for smaller a/t ratios Figure 12a–f show an initial increase and subsequent plateauing in $K_{I}$ values as a/2c increases, which is similar to the upper-bound $K_{I}$ trends observed in Figure 11 except for the higher a/t, where a post-peak decline in $K_{I}$ values for higher R/a ratios is accentuated. This similarity indicates that the initial stress response and peak stress characteristics are consistent between the upper and lower bounds of stress intensity across these configurations.

### 4.3. Correlation Analysis

Correlation analysis is a statistical method used to measure the strength and direction of the relationship between variables. In the context of fracture mechanics, this analysis is applied to investigate the influence of geometric parameters on the $K_{I}$ value. However, employing a direct correlation between $K_{I}$ and geometric parameters could lead to an overestimation of the correlation coefficients. This is primarily because $K_{I}$ is directly proportional to the square root of the crack size (R), which can skew the correlation if not appropriately normalized. To address this issue and ensure a more accurate analysis, the shape factor (β) is used for the correlation analysis. The $K_{I}$ value is normalized by dividing $K_{I}$ by the stress and the square root of π times the crack radius.

(2) $$\beta \;=\;\frac{K_{I}}{\sigma \;\sqrt{\pi \;R}}$$

The most common measure of correlation is the Pearson correlation coefficient (often denoted as $r_{XY})$. It quantifies the linear correlation between two datasets. Its value ranges from −1 to 1, where:

- $r_{XY}=$ 1 indicates a perfect positive linear relationship;
- $r_{XY}=$ −1 indicates a perfect negative linear relationship;
- $r_{XY}=$ 0 indicates no linear relationship.

To measure the linear correlation between the derived ratios (a/2c, R/a, and a/t) and the $K_{I}$, the Pearson correlation coefficient is defined as:

(3) $$r_{XY}=\frac{\sum \left(X_{i}-\bar{X}\right)\left(Y_{i}-\bar{Y}\right)}{\sqrt{\sum {\left(X_{i}-\bar{X}\right)}^{2}\sum {\left(Y_{i}-\bar{Y}\right)}^{2}}}$$

where $\left(X_{i}\right)$ and $\left(Y_{i}\right)$ are individual sample points of the dimensionless parameters and $K_{I}$, respectively, and $\left(\bar{X}\right)$ and $\left(\bar{Y}\right)$ are the mean values of (X) and (Y), respectively.

**Figure 13:**
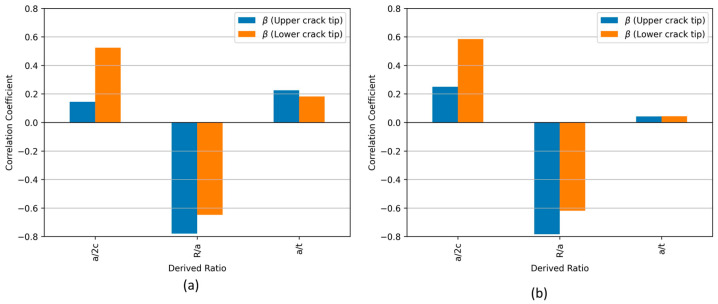
(**a**) Correlation of normalized geometric parameters with β at both crack tips. (**b**) Adjusted correlation analysis excluding two specific cases with a/t = 0.3 and 0.5.

Figure 13a shows the correlation coefficients between different geometric parameters and the β factor for both the upper and lower crack tips. The normalized geometric parameter R/a shows a strong negative correlation coefficient with β for both crack tips. When comparing the effect of R/a for the upper and lower crack tips, the results show a greater impact for the upper crack tip.

In Figure 13a, which includes all cases, a/t exhibits a positive correlation with β for both the upper and lower crack tips. However, after excluding the thin plate cases in Figure 13b, the correlation coefficient for a/t decreases significantly, approaching zero. This reduction suggests that for thicker plates under tensile loading, the influence of a/t on β becomes negligible. The weakening of the correlation implies that for thick walls, the effect of a/t is minimal in determining the value of β, indicating that other geometric parameters dominate the behaviour of crack-tip fields in these conditions.

The parameter a/2c exhibits positive correlations with β. The correlations suggest that a/2c has a complex effect on β values, with a much greater impact on the lower crack tip as compared to the upper crack tip. The positive correlation of β with a/2c for the upper crack tip is less when the thin plates are included in the correlation analysis. This is further observed in Figure 13b for a/t values lower than 0.1 (thick plates). This is confirmed by the results presented in Figure 11e,f, where a decline in $K_{I}$ can be observed for a/t ratios higher than 0.1.

### 4.4. Surrogate Model for the Stress Intensity Factor $K_{I}$

The data obtained from the FEA (216 simulations) are incorporated into a predictive model using a k-dimensional (k-d) tree. The k-d tree algorithm is a space-partitioning method used to organize points in a k-dimensional space, facilitating efficient searches by including nearest-neighbour searches. The construction of a k-d tree involves recursively dividing the dataset into two subsets along one of the k dimensions using the median of the points, alternating the dimension at each level of the tree. This division creates a binary tree where each node represents a point in the k-dimensional space, and the left and right subtrees contain points that are, respectively, less than or equal to, and greater than the node’s point along the chosen dimension [38]. It is used for pattern recognition, data mining, financial market predictions, intrusion detection, and more.

**Figure 14:**
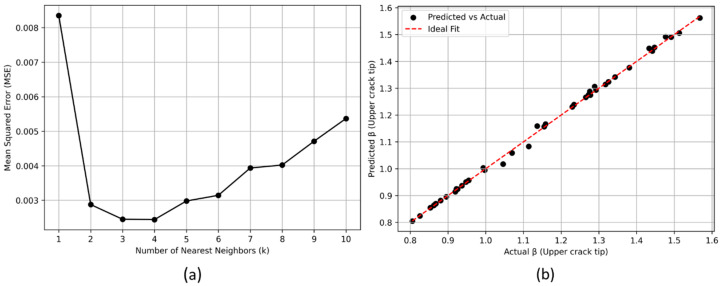
(**a**) Mean squared error (MSE) for different values of the number of nearest neighbours (k); (**b**) Actual vs. predicted values of β (upper crack tip) using the k-NN model for k = 4.

When a set of raw input parameters (a,2c,R,t) is provided, these are first converted to normalized parameters (a/2c, R/a, a/t). These normalized parameters are used to construct and query the k-d tree. The k-NN (k-Nearest Neighbour) algorithm is then used to find the nearest neighbours to a given query point. The inverse weighting distance concept is then used to calculate the β value based on the values of the nearest neighbours. The weights assigned to each value are inversely proportional to the distances from the query point. This means that closer neighbours have higher weights, giving them more influence on the interpolated value. The optimal number of neighbours depends on the dataset and the desired balance between bias and variance. In order to determine the optimal value, the entire dataset is split into training and validation sets using an 80–20 split. This split allows to train the model on one subset of the data and validate its performance on an unseen subset, ensuring that the model’s evaluation is unbiased. The performance is evaluated for different values of k (ranging from 1 to 10) by calculating the mean squared error (MSE) between the predicted and actual β. The MSE values for different k values are plotted in Figure 14a to visualize the convergence. The optimal k is found to be four for this dataset. Using this value, predictions are made using the validation set. A scatter plot is shown in Figure 14b to compare the actual β values with the predicted values.

**Figure 15:**
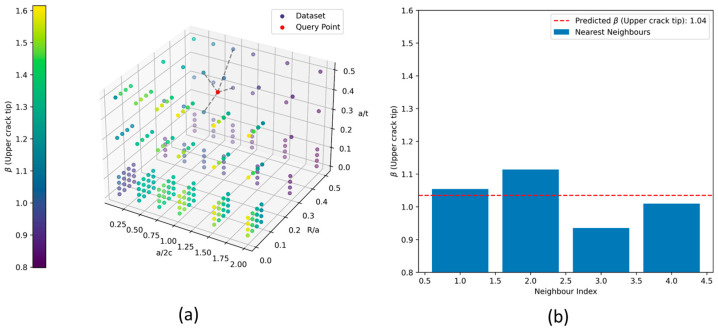
(**a**) An example of a k-d tree nearest neighbour structured in 3D for shape values at the upper crack tip, and (**b**) predicted β value using the nearest neighbour approach.

The 3D scatter plot in Figure 15a visualizes the spatial distribution of the dataset in a three-dimensional parameter space defined by the normalized variables $\left(a/2c,R/a,a/t\right)$. Each coloured sphere represents a single data point, with the colour indicating the β value for the upper crack tip at that point. The red star in the plot marks the query point, which corresponds to a specific set of crack parameters $\left(a/2c=0.8,\;R/a=0.35,a/t=0.38\right)$. This point is where the β value is predicted using the k-NN approach. The dashed grey lines connect the query point to its four nearest neighbours.

In Figure 15b, the bar plot shows the β values for the four nearest neighbours and the predicted β value for the query point. The red dashed line in the plot represents the predicted β value for the query point, calculated using inverse distance weighting of the β values from the nearest neighbours. The predicted β value is then used to determine the $K_{I}$ based on the input stress value. A GUI is created to provide an efficient means of $K_{I}$ calculations. This Python code can be found in the public repository (https://doi.org/10.17605/OSF.IO/J79MH) under the ‘GUI LUT’ directory.

The choice of six points for each geometric parameter range appears to be a valid approach, as evidenced in Section 4.1 and Section 4.2 of this study. The trends observed in Figure 11 and Figure 12 clearly show peaks and plateauing in the varying geometric parameters, indicating that this choice was suitable for capturing the effects of geometric parameters. Furthermore, the use of this dataset in the k-NN surrogate model has yielded predictions with high accuracy as shown in Figure 14b, demonstrating that the chosen points were not only sufficient to capture the underlying effects but also contributory in constructing a reliable predictive model.

## 5. Conclusions

In this study, a comprehensive parametric investigation of Mode I stress intensity factor ($K_{I}$) for cracks emanating from semi-ellipsoidal pits under uniaxial tensile stress was conducted using a finite element analysis. The analysis focused on the influence of normalized geometric parameters, namely pit-depth-to-pit-width (a/2c), pit-depth-to-plate-thickness (a/t), and crack-radius-to-pit-depth (R/a). Some studies investigated the $K_{I}$ of mouth-initiated cracks, but those reports remain limited to solutions for a few pits.

To facilitate efficient and accurate KI calculations, an automated framework integrating Python scripts with ABAQUS^®^ CAE 2023 was developed. This framework streamlined the pre- and post-processing steps, ensuring high accuracy and reduced computational time. Our investigation involved 216 simulations, revealing several key insights:

- Pit Depth-to-Width Ratio (a/2c): Deeper pits (higher a/2c ratios) exhibit significantly higher stress concentrations at the crack tips. For instance, when a/2c increased from 0.1 to 1.0, $K_{I}$ values increased considerably, indicating more severe stress intensity near deeper pits. However, beyond an a/2c ratio of 1.0, the increase in $K_{I}$ tended to stabilize or slightly decrease, particularly for smaller R/a values.
- Crack Radius-to-Pit Depth Ratio (R/a): The R/a ratio is identified as the most influential parameter. $K_{I}$ values increased markedly with higher R/a ratios. For example, an increase in R/a from 0.01 to 0.5 resulted in a substantial rise in $K_{I}$, emphasizing that larger cracks relative to pit depth led to higher stress intensity factors.
- Pit Depth-to-Plate Thickness Ratio (a/t): Higher a/t ratios led to a pronounced increase in $K_{I}$ values. In scenarios with a/t ratios above 0.1, the $K_{I}$ values initially increased with higher a/2c ratios but eventually showed a declining trend as a/2c continued to rise.

A surrogate model for $K_{I}$ values is constructed using a k-dimensional tree algorithm, enabling a prediction of $K_{I}$ for various pit configurations. The model uses normalized geometric parameters (a/2c, R/a, a/t) to predict β values based on inverse distance weighting of the nearest neighbours. A user-friendly GUI was created to provide an efficient means of $K_{I}$ calculations, utilizing the developed surrogate model.

## Figures and Tables

**Table 1:** Comparison of displacement extrapolation and J-integral methods for SIF calculation using 3D FEM.

Feature	Displacement Extrapolation Method	J-Integral Method
Flexibility with element types	Works with various element types, including tetrahedral and hexahedral, offering versatility in modelling complex geometries.	Requires hexahedral elements for the contour path, which can be challenging in complex geometries.
Complex geometries	Well-suited for analyses involving intricate geometrical configurations.	Setting up a J-integral calculation can be complex, especially for models with intricate geometries.
Mesh sensitivity	Highly dependent on the quality of the mesh near the crack tip, requiring careful mesh refinement.	Less affected by the meshing directly at the crack tip due to the integral path being away from the singularity.
Calculation approach	Relies on extrapolating displacements from nodes to the crack tip, which can introduce errors.	Integrates values around a path encircling the crack tip, providing a direct approach to SIF calculation.
Implementation complexity	May require additional calculations for extrapolation, which can be time consuming.	Built-in capabilities in many FEA software packages simplify the process, although mesh requirements at integral paths may pose challenges.
Accuracy	Accuracy is influenced by the near-tip mesh refinement.	Theoretically path independent, offering potentially more accurate SIF calculations without requiring refined mesh near the tip.

**Table 2:** Simulation matrix.

Parameters	Ranges
a/2c	0.1, 0.4, 0.7, 1.0, 1.5 and 2.0
R/a	0.01, 0.04, 0.07, 0.1, 0.3 and 0.5
a/t	0.01, 0.04, 0.07, 0.1, 0.3 and 0.5

## Data Availability

The database mentioned in this article is published in an open-access repository. It can be accessed with the following link: https://doi.org/10.17605/OSF.IO/J79MH.
